# Dabrafenib‐Induced Cutaneous Eruptions in a Patient With Langerhans Cell Histiocytosis

**DOI:** 10.1155/crdm/5004676

**Published:** 2026-07-14

**Authors:** Jaanvi Mehta, Julianna Tolotta, Eleanor Matthews, Daniel Joffe, Safiyyah Bhatti, Lauren Banner, Jenna Mandel, Molly Wallace, Timothy Webster, Jason B. Lee, Neda Nikbakht

**Affiliations:** ^1^ Sidney Kimmel Medical College, Thomas Jefferson University, Philadelphia, Pennsylvania, USA, jefferson.edu; ^2^ Department of Dermatology & Cutaneous Biology, Thomas Jefferson University, Philadelphia, Pennsylvania, USA, jefferson.edu; ^3^ Department of Hematology & Oncology, Thomas Jefferson University, Philadelphia, Pennsylvania, USA, jefferson.edu

## Abstract

Langerhans cell histiocytosis (LCH) is a rare malignancy marked by clonal proliferation of Langerhans cells, with *BRAF* V600E mutations identified in over 50% of the cases. BRAF inhibitors (BRAFi), such as dabrafenib, have shown efficacy in treating *BRAF* V600E‐mutant LCH. However, BRAFi therapy is associated with cutaneous adverse effects, including the development of new melanocytic nevi, verrucae, and keratinocyte carcinoma (KC). This case report describes a 78‐year‐old woman with *BRAF* V600E‐mutant LCH who developed multiple dermatologic side effects following dabrafenib salvage therapy. Seven weeks into treatment, the patient presented with eruptive palmoplantar nevi, verrucae, and a basal cell carcinoma (BCC). Biopsies revealed endophytic verruca vulgaris and acral junctional melanocytic nevus. A previous history of KC and photodamage likely contributed to the development of BCC. Additionally, the patient experienced arthralgias and Dupuytren’s contracture, consistent with known BRAFi side effects. While the cutaneous manifestations observed here have been documented in *BRAF* V600E‐mutant melanoma, this case is unique in its presentation in LCH patients. This report emphasizes the need for routine dermatologic monitoring and awareness of potential skin malignancies and other side effects in adults undergoing BRAFi therapy for LCH.

## 1. Introduction

Langerhans cell histiocytosis (LCH) is a rare malignancy characterized by pathologic clonal proliferation of Langerhans cells in various anatomic sites such as skin, bone, spleen, and liver. Langerhans cells are antigen‐presenting cells of dendritic origin that reside in the skin [1]. Molecular analysis reveals that over 50% of the patients with LCH have a *BRAF* V600E mutation, making BRAF kinase a therapeutic target in the treatment of *BRAF* V600E‐mutant LCH [2]. BRAF kinase is a key enzyme in the mitogen‐activated protein kinase (*MAPK*) signaling cascade [3]. The Food and Drug Administration has approved inhibitors of BRAF kinase (BRAFi) for the treatment of *BRAF* V600E‐mutant malignancies, such as LCH [4]. BRAFi have shown efficacy treating *BRAF* V600E‐mutant LCH, both as first‐line and salvage therapy [2, 5, 6]. BRAFi are associated with several cutaneous adverse effects (cAEs) including the development of new melanocytic nevi, increased size and pigmentation of existing nevi, and an increased risk of keratinocyte carcinoma (KC) [3]. Here we present a case report describing an adult patient with LCH who developed cAEs, including palmoplantar nevi and verrucae, in the setting of BRAFi salvage therapy.

## 2. Case Presentation

A 78‐year‐old Caucasian woman with a past medical history of chronic obstructive pulmonary disease, KC, and *BRAF* V600E‐mutant LCH presented to the dermatology office with multiple cutaneous lesions. Prior to presentation, the patient was initiated on 150 mg dabrafenib salvage therapy for *BRAF* V600E‐mutant multisystem LCH refractory to cytarabine.

The patient’s oncologic history is as follows. Approximately 2 years prior to the development of cAEs, the patient presented to the pulmonologist with abdominal and lower back pain, fatigue, unintentional weight loss, and dysgeusia. She was admitted to the hospital where lab workup revealed anemia and thrombocytopenia, and an abdominal CT scan revealed splenomegaly. The patient underwent a diagnostic splenectomy. Pathology and molecular analysis of the spleen were diagnostic of *BRAF* V600E‐mutant multisystem LCH. Positron emission tomography‐computed tomography (PET‐CT) imaging revealed hypermetabolic osseous metastases involving lumbar vertebrae (L4 standardized uptake value (SUV) 6.47), the right iliac wing (SUV 2.78), the intertrochanteric right femur (SUV 3.13), and the ischial bones (right SUV 5.37 and left SUV 2.69). Following diagnosis, the patient underwent 12 cycles of cytarabine therapy with complete therapeutic response noted with a PET‐CT scan performed at treatment conclusion. For comparison, the lytic lesion of the right femur had an SUV of 1.7 after treatment. Restaging evaluation with PET‐CT scan 6 months after completion of cytarabine therapy revealed LCH recurrence, with FDG‐avid malignancy involving the bilateral lungs (SUV 4.1 in the left and 3.0 in the right), and extensive active bony lesions of the spinal column, ribs, and pelvic bones (SUV 15.1 at L1 vertebral body, SUV 10.6 at T8 vertebral body, and SUV 11.4 at L4 vertebral body). At this time, salvage therapy was initiated with dabrafenib 150 mg twice daily (corresponding to Day 0). The dose was decreased to 75 mg daily at Day 114 after restaging PET‐CT performed four months later revealed complete response, with a Deauville score of 1.

**FIGURE 1:**
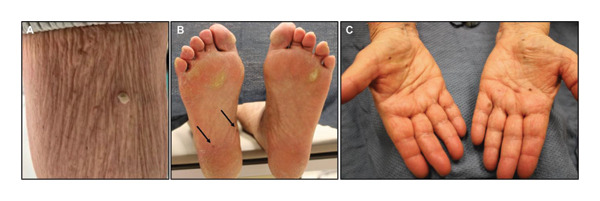
Clinical findings. (A) Exophytic hyperkeratotic lesion on patient’s left calf diagnosed as verruca vulgaris. (B) Plantar melanocytic nevi (arrows) and plantar hyperkeratotic calluses. (C) Palmar melanocytic nevi.

**FIGURE 2:**
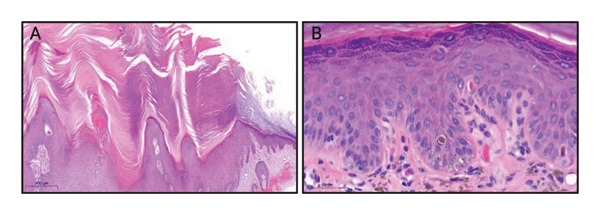
Histopathologic findings. (A) Histopathologic findings of endophytic verruca vulgaris on left calf. There is hyperkeratosis, acanthosis, and papillomatosis (hematoxylin & eosin stain, magnification 3x). (B) Histopathologic findings of acral junctional melanocytic nevus on left palmar surface. There are small, discrete nests of monomorphic melanocytes at the dermoepidermal junction with minimal pagetoid scatter of melanocytes, papillary dermis with many melanophages, and discrete, thin columns of melanin in the cornified layer (hematoxylin & eosin stain, magnification 40x).

Skin exam on Day 23 revealed multiple exophytic hyperkeratotic lesions scattered throughout the body, most notably on the left calf (Figure 1(A)) and on the soles of the feet (Figure 1(B)). Multiple dark brown to black macules with globules in ridges were apparent on the palmar and plantar surfaces (Figures 1(B) and 1(C)) on repeat examination on Day 163. The patient also presented with a telangiectatic pink papule on the right cheek, noted on Day 338. Shave biopsy of the hyperkeratotic lesion on the left calf was consistent with endophytic verruca vulgaris (Figure 2(A)) and shave biopsy of a dark macule on the left palm revealed an acral junctional melanocytic nevus (Figure 2(B)). Biopsy of the pink papule on the patient’s cheek revealed basal cell carcinoma (BCC). The patient’s hyperkeratotic plantar lesions were treated with 40% urea cream. The patient was reassured that the melanocytic nevi were benign, and she was scheduled for Mohs surgery for removal of the BCC. The patient continues to be monitored in the dermatology clinic every 8 weeks.

Throughout the course of dabrafenib salvage treatment, the patient experienced diffuse arthralgias that improved following dose reduction. The patient also developed discomfort closing the left hand that was diagnosed as a Dupuytren’s contracture.

## 3. Discussion

This case represents numerous cAEs following dabrafenib therapy for *BRAF* V600E‐mutant LCH, including eruptive nevi in a unique palmoplantar distribution, verrucae, and KC. There is limited literature describing cAEs of BRAFi therapy for adult LCH patients, with previous literature describing the development of melanoma, maculopapular rash [2], and diffuse hyperkeratotic lesions [7]. To our knowledge, no other dabrafenib‐associated cAEs have been documented in adults with LCH. Melanocytic nevi and maculopapular rash have been described in dabrafenib‐treated pediatric LCH patients [6]. Prior studies have characterized in detail cutaneous findings following BRAFi therapy for other malignancies. For instance, one patient with papillary thyroid cancer treated with vemurafenib developed acral nevi [8]. Following encorafenib treatment for colorectal cancer, the development of new nevi, including in a palmoplantar distribution [9], and KA‐type SCC have been described [10]. Reported adverse effects following BRAFi therapy for *BRAF* V600E‐mutant melanoma include the development of new melanomas, KC, NMSCs, hyperkeratotic lesions, new melanocytic nevi, and morphological changes to previously existing nevi [3]. Dupuytren’s contracture following BRAFi therapy is a notable noncutaneous adverse effect that has also been documented in the setting of *BRAF* V600E‐mutant melanoma [11].

LCH predominantly affects the pediatric population with an incidence of 8.9 cases per million per year, in comparison to an incidence of 0.07 cases per million per year in the adult population. Current therapies for LCH include surgical resection of isolated affected areas, or chemotherapy and radiation for more extensive involvement [1]. Unfortunately, treatment failure is not uncommon, as approximately 50% of the patients develop relapsed or refractory LCH within 5 years of initial therapy [5].

Paradoxical reactivation of *MAPK* signaling is thought to underlie the hyperproliferative cutaneous and noncutaneous adverse effects associated with the use of BRAFi [7, 8, 12]. Following BRAFi therapy, increased *MAPK* signaling has been observed in cells lacking the *BRAF* V600E mutation and produces downstream outcomes such as increased cell proliferation and survival. This mechanism is thought to underlie the development of cAEs following BRAFi therapy [3], with increased proliferation of *BRAF* wild‐type melanocytes resulting in the development of nevi, and proliferation of BRAF wild‐type keratinocytes contributing to the formation of verrucae [12, 13].

Dupuytren’s contractures are also proposed to develop because of increased *MAPK* signaling [11].

*MAPK* pathway reactivation in epidermal cells with activating *RAS* mutations induced by ultraviolet (UV) light damage may explain the development of malignant cAEs. Patients with a history of photodamage initiated on BRAFi may, therefore, be predisposed to developing cutaneous malignancy [8, 12]. One study found that adults treated with BRAFi for *BRAF* V600E‐mutant metastatic melanoma developed KCs following therapy [6]. KCs have not been observed in pediatric patients treated with BRAFi, perhaps because children have less photodamage and, therefore, have fewer keratinocytes harboring UV‐induced RAS mutations [12]. In the case described here, the patient’s likely history of photodamage contributing to prior KCs may have predisposed her to BCC development. Alongside chronic sun exposure, other factors that may contribute to increased photodamage, such as germline mutations, may be considered when evaluating susceptibility to cutaneous malignancy [14].

As BRAFi are more widely utilized in the treatment of LCH, it is important to monitor and study cAEs that may be consequent to BRAFi in LCH patients. Our case highlights the importance of routine skin examinations and long‐term monitoring of adult patients undergoing BRAFi therapy due to the potential for cutaneous eruptions and malignancy. A causal relationship between dabrafenib initiation and cAE onset cannot be determined from a single case. Larger case series and pharmacovigilance analyses are warranted to characterize BRAFi‐associated cAEs in LCH patients.

**Nomenclature**
LCH: Langerhans cell histiocytosis
MAPK: Mitogen‐activated protein kinase
BRAFi: BRAF inhibitor
KC: Keratinocyte carcinoma
BCC: Basal cell carcinoma
SUV: Standardized uptake value
cAE: Cutaneous adverse effect
PET‐CT: Positron emission tomography‐computed tomography
UV: Ultraviolet

## Author Contributions

Jaanvi Mehta: writing–original draft, writing–review and editing, and visualization.

Julianna Tolotta: writing–original draft, writing–review and editing, and visualization.

Eleanor Matthews: writing–original draft.

Daniel Joffe: writing–review and editing, visualization, and conceptualization.

Safiyyah Bhatti: writing–review and editing.

Lauren Banner: writing–review and editing.

Jenna Mandel: writing–review and editing.

Molly Wallace: writing–review and editing.

Timothy Webster: conceptualization.

Jason B. Lee: writing–review and editing, supervision, and conceptualization.

Neda Nikbakht: writing–review and editing, supervision, and conceptualization.

## Funding

The authors have nothing to report.

## Disclosure

The authors declare no conflicts of interest.

## Consent

The authors obtained written consent from patients for their photographs and medical information to be published in print and online and with the understanding that this information may be publicly available. Patient consent forms were not provided to the journal but are retained by the authors.

## Conflicts of Interest

The authors declare no conflicts of interest.

## Data Availability

Data sharing not applicable to this article as no datasets were generated or analyzed during the current study.
